# Endometrial Hyperplasia: Current Insights into Epidemiology, Risk Factors, and Clinical Management

**DOI:** 10.3390/cancers18010148

**Published:** 2025-12-31

**Authors:** Apostolia Galani, Sofoklis Stavros, Efthalia Moustakli, Anastasios Potiris, Athanasios Zikopoulos, Ismini Anagnostaki, Konstantinos Zacharis, Maria Paraskevaidi, Deirdre Lyons, Stefania Maneta-Stavrakaki, Nikolaos Thomakos, Maria Kyrgiou, Ekaterini Domali

**Affiliations:** 1 Department of Metabolism, Digestion and Reproduction, Faculty of Medicine, Imperial College London, London W12 ONN, UK; l.galani22@imperial.ac.uk (A.G.); m.paraskevaidi@imperial.ac.uk (M.P.); deirdre.lyons2@nhs.net (D.L.); s.maneta-stavrakaki17@imperial.ac.uk (S.M.-S.); m.kyrgiou@imperial.ac.uk (M.K.); 2 Third Department of Obstetrics and Gynecology, University General Hospital “ATTIKON”, Medical School, National and Kapodistrian University of Athens, 12462 Athens, Greece; 3 Department of Nursing, School of Health Sciences, University of Ioannina, 4th kilometer National Highway Str. Ioannina-Athens, 45500 Ioannina, Greece; ef.moustakli@uoi.gr; 4 Torbay and South Devon NHS Foundation Trust Lowes Brg, Torquay TQ2 7AA, UK; thanzik92@gmail.com; 5 Medical School, National and Kapodistrian University of Athens, 11527 Athens, Greece; isanagnostaki3@gmail.com; 6 Department of Obstetrics and Gynecology, General Hospital of Lamia, 35100 Lamia, Greece; zaxarisk@yahoo.com; 7 First Department of Obstetrics and Gynecology, Alexandra Hospital, Medical School, National and Kapodistrian University of Athens, 11528 Athens, Greece; thomakir@hotmail.com (N.T.); kdomali@yahoo.fr (E.D.)

**Keywords:** PCOS, metabolic dysfunction, AUB, biomarkers, progestin therapy

## Abstract

Endometrial hyperplasia (EH) comprises a spectrum of abnormal proliferative changes in the endometrium, ranging from benign glandular overgrowth to lesions with substantial malignant potential. The importance of risk stratification and early identification is highlighted by the growing recognition of EH as a precursor to endometrial cancer. The main causes of EH, according to epidemiological research, include obesity, polycystic ovarian syndrome (PCOS), metabolic dysfunction, and extended exposure to unopposed estrogen. Emerging molecular markers, histological analysis, and imaging are all necessary for a proper diagnosis of EH because it might appear with vague clinical symptoms such as irregular uterine bleeding. Surgical intervention or progestin therapy are two possible management techniques for EH, depending on the lesion’s intricacy and the patient’s medical history, including fertility issues. Personalized therapy techniques and recent developments in molecular profiling have the potential to enhance patient outcomes by matching treatment to tumor biology and individual risk profiles. This review highlights the translational potential of molecular insights while synthesizing the most recent data on the epidemiology, risk factors, diagnostic techniques, and therapy of EH. A deeper comprehension of these elements is necessary to maximize treatment results and stop the development of endometrial cancer.

## Simple Summary

Endometrial hyperplasia (EH) is a condition where the lining of the uterus grows too much, sometimes leading to endometrial cancer if not treated. Common risk factors include obesity, polycystic ovarian syndrome, metabolic problems, and long-term exposure to estrogen without progesterone. EH can cause symptoms like irregular bleeding, but sometimes it is found by chance. Diagnosis usually combines imaging, tissue sampling, and sometimes molecular tests. Treatment can involve hormonal therapy with progestins or surgery, depending on how severe the condition is and whether the patient wants to keep fertility. New research using molecular profiling is helping doctors personalize treatment and better predict which cases might progress to cancer. Understanding risk factors, early diagnosis, and individualized care are key to preventing endometrial cancer and improving patient outcomes.

## 1. Introduction

Histologically, endometrial hyperplasia (EH) encompasses a spectrum of non-invasive proliferative disorders characterized by an increased gland-to-stroma ratio relative to normal endometrium. Importantly, EH is not a single pathological entity; rather, it includes both hyperplasia without atypia, which carries a low malignant potential, and premalignant lesions such as atypical hyperplasia (AH) and endometrial intraepithelial neoplasia (EIN) [1,2]. Throughout this review, we use the term “endometrial hyperplasia (EH)” as an umbrella term encompassing hyperplasia without atypia, atypical hyperplasia, and EIN. However, these entities differ substantially in malignant potential and management, any discussion of progression to cancer refers specifically to atypical hyperplasia/EIN unless otherwise stated.

Although, AH and EIN have often been used interchangeably, they are not fully synonymous. EIN represents a biologically and morphometrically defined clonal premalignant lesion, established through criteria developed by Mutter and the Endometrial Collaborative Group [3]. However, according to the previous WHO 1994 paradigm, AH is a solely histomorphologic diagnosis based on cytologic atypia. Despite significant clinical behavior overlap, EIN offers better repeatability and more correctly predicts the development of endometrioid cancer [4].

Historically, EH was classified into four types: simple hyperplasia, complicated hyperplasia, and each with or without atypia. The World Health Organization (WHO) system streamlined this into two groups due to limited clinical utility and poor reproducibility: (i) non-atypical hyperplasia, associated with a low risk of malignant transformation (approximately 1–3%), and (ii) atypical hyperplasia/EIN, which has a significantly higher risk of progression (25–40% if left untreated [1,5].

Interpreting reported progression risks requires caution. Much of the available evidence on atypical hyperplasia/EIN progression is limited by the difficulty to distinguish true progression from pre-existing, but unsampled, endometrial cancer. Early studies including Kurman et al. (1985) reported progression rates of ~23%, yet many diagnoses were based on dilation and curettage (D&C) or office biopsy, methods which may fail to detect concurrent cancer [6]. More recent long-term estimates, such as the 20-year progression risk of 28% reported by Lacey et al., are subject to similar methodological constraints. Because endometrial sampling may miss focal malignant areas, especially with office-based biopsy, published progression estimates likely reflect a combination of true progression and initially occult carcinoma [7].

The current WHO 2014 classification merges AH and EIN into a single category—atypical hyperplasia/EIN—because both identify lesions with significantly increased malignant potential. Accordingly, throughout this review, we use the term EH to refer broadly to the full hyperplasia spectrum and distinguish non-atypical hyperplasia from atypical hyperplasia/EIN, the latter representing the biologically premalignant group [8]. Since diagnostic accuracy depends on tissue sampling, it is important to note that office-based endometrial biopsy (e.g., Pipelle) may miss focal lesions, D&G provides a broader but still blind sampling, and hysteroscopy-guided biopsy offers the highest accuracy through direct visualization.

EH development is closely associated with several recognized risk factors, many of which overlap with those for endometrial cancer [9]. Obesity-related peripheral conversion of androgens, prolonged anovulation, estrogen-secreting tumors, and estrogen-only hormone replacement treatment are the main causes of unopposed estrogen exposure. Obesity is particularly significant given its global prevalence and its dual role in increasing estrogen levels and promoting insulin resistance [10,11].

Additional risk factors include nulliparity, early menarche, late menopause, diabetes mellitus, metabolic syndrome, tamoxifen therapy, polycystic ovary syndrome (PCOS), and genetic cancer syndromes, including Lynch syndrome [12,13]. These factors converge to create a prolonged estrogen-dominant condition that stimulates endometrial proliferation, inhibits apoptosis, and promotes genetic alterations that may progress to atypical hyperplasia or cancer [14].

Molecular research has further expanded our understanding of EH pathophysiology. Identification of molecular biomarkers has the potential to refine risk stratification, enhance diagnostic accuracy, and guide targeted therapies [15,16,17]. Recent large-scale molecular profiling investigations and prospective treatment meta-analyses have made it clear which conservative treatments result in long-lasting remission and which EH lesions have the highest propensity for malignancy [18,19,20]. This manuscript synthesizes (i) new longitudinal risk estimates from cohort studies, (ii) advances in molecular stratification (integration of PTEN/PIK3CA/KRAS alterations with immunohistochemical surrogates like MMR and p53), and (iii) randomized and comparative data favoring the levonorgestrel intrauterine system (LNG-IUS) over oral progestins for many non-atypical lesions, in contrast to previous descriptive reviews. We identify gaps for routine molecular integration into practice and highlight areas where new information changes clinical decision criteria compared to earlier guidelines.

As obesity, metabolic syndrome, and reproductive endocrine disorders rise globally, understanding the epidemiology, risk factors, and clinical implications of EH is increasingly important. This review aims to identify at-risk populations and promote prevention, early detection, and evidence-based management by synthesizing the most recent and clinically relevant research on EH. Furthermore, this review integrates emerging molecular, metabolic, and clinical data to illustrate how these factors converge to influence disease progression and therapeutic response. This conceptual synthesis distinguishes the present review from earlier summaries and underscores its contribution to advancing individualized risk assessment and management strategies.

## 2. Methodology

A comprehensive literature search was performed to identify studies relevant to EH, including its risk factors, diagnosis, imaging modalities, molecular markers, and management strategies. PubMed, Embase, and Scopus were searched for articles using combinations of keywords such as “endometrial hyperplasia”, “endometrial cancer precursor”, “PCOS”, “metabolic dysfunction”, “progestin therapy”, “imaging”, and “biomarkers”. Only articles published in English were considered. Titles and abstracts were screened for relevance, followed by full-text review. Given the narrative nature of this review, studies were selected to provide a broad and comprehensive overview of current knowledge rather than a systematic or quantitative synthesis.

## 3. Epidemiology

EH is a relatively common gynecologic condition, particularly among women reporting abnormal uterine bleeding (AUB) in the perimenopausal and early postmenopausal years [21]. Although prevalence varies according to diagnostic criteria, healthcare access, and population characteristics, the incidence in high-income counties is estimated at approximately 133 cases per 100,000 women per year. EH is identified in 10–20% of women undergoing endometrial biopsies for AUB, while atypical hyperplasia accounts for 1–2% of cases [22,23].

The risk of EH increases with age and peaks in the fifth and sixth decades of life, when anovulatory cycles become more frequent and progesterone levels decline [24]. EH is less common in younger women because regular ovulatory cycles typically provide sufficient progesterone to counterbalance the proliferative effects of estrogen on the endometrium. Nevertheless, EH can occur at any reproductive age, especially among those with obesity, PCOS, or chronic anovulation, which are conditions associated with prolonged unopposed estrogen exposure. In postmenopausal women, EH most often results from endogenous estrogen excess, including estrogen-producing tumors such as granulosa cell tumors or obesity-related peripheral aromatization. The use of unopposed estrogen therapy has become uncommon due to routine endometrial protection with progestogens [25,26,27].

Atypical hyperplasia/EIN is the most clinically significant form of EH because of its malignant potential [28]. In a large cohort study by Lacey et al., the 20-year risk of progression to endometrial cancer was 27.5% for atypical hyperplasia, compared to non-atypical hyperplasia which was less than 5% [29]. Diagnostic limitations of endometrial sampling are underscored by the finding that approximately 30–45% of women with atypical hyperplasia are found to have concurrent cancer at the time of hysterectomy, suggesting a substantial burden of subclinical diseases [28,30,31].

There have been reports of differences in EH prevalence by geography and ethnicity. Similarly to higher prevalence of obesity and metabolic syndrome, higher rates are seen in North America and Northern Europe. Parts of Asia and Africa, on the other hand, have lower prevalence; however, these disparities seem to be closing as diet and lifestyle become more westernized [32,33,34].

Furthermore, epidemiological statistics indicate that the incidence of EH is increasing globally, most likely due to increased use of estrogen-containing medications, obesity, delayed childbearing, and decreased parity [35]. The lack of adequate diagnostic and treatment resources in low- and middle-income countries (LMICs) makes this development particularly worrisome because it may allow cancer progression to go unnoticed [36].

Since endometrial cancer is mostly caused by EH, tracking its epidemiological trends offers important information about the future incidence of gynecologic cancer [37]. Reducing the progression of EH to malignancy requires increased awareness, prompt diagnosis, and proactive management, especially in high-risk population [38].

## 4. Risk Factors

A complex interplay of hormonal, metabolic, genetic, and environmental factors that contributes to the development of EH. Understanding of these risk profiles is essential for prevention, early detection, and personalized treatment strategies [39].

### 4.1. Hormonal Influences

The endometrium is extremely susceptible to changes in sex steroid hormones. The most significant risk factor for the development of EH remains the prolonged or excessive exposure to unopposed estrogen, whether it comes from an endogenous or exogenous source [2]. Estrogen promotes the growth of endometrial glands, while progesterone inhibits mitotic activity and induces secretory differentiation. Inadequate progesterone therefore allows continuous, unregulated proliferation that predisposes to hyperplastic change [40].

This hormonal imbalance is best illustrated by conditions like PCOS, which are linked to persistent anovulation or progesterone deficiency [41,42]. Exogenous exposures can also contribute; ovarian tumors that secrete estrogen, long-term usage of selective estrogen receptor modulators (like tamoxifen), and hormone replacement therapy with estrogen alone are all known to contribute [43].

Lifetime estrogen exposure is considerably altered by reproductive history. Nulliparity, late menopause, and early menarche increase risk by extending the duration of unopposed estrogen action [44]. However, combined oral contraceptives and multiparity provide protection through cyclic or prolonged progesterone dominance [45].

Understanding these mechanisms directly informs prevention and management, include the use of progestin therapy for non-atypical EH, ovulation induction in PCOS, and avoidance of unopposed estrogen in postmenopausal hormone therapy.

### 4.2. Metabolic Factors

Metabolic dysfunction plays a major role in EH pathogenesis. The most important metabolic risk factor is obesity [46]. Excess adipose tissue aromatization of androgens to estrogens contribute to persistently elevated estrogen levels beyond normal physiological conversion [47]. Furthermore, reduced levels of sex hormone-binding globulin (SHBG) further increase bioavailable estrogen [48].

Beyond the effects of estrogen, insulin resistance and hyperinsulinemia linked to obesity intensify mitogenic signaling pathways in the endometrium, which encourages aberrant proliferation. Through processes such as systemic inflammation, insulin-like growth factor (IGF) activation, and chronic hyperinsulinemia, diabetes mellitus also contributes, even when it is not associated with obesity [49,50].

Metabolic syndrome, characterized by central obesity, hypertension, dyslipidemia, and insulin resistance, creates a pro-inflammatory and pro-hormonal environment that favors EH development and progression [51]. Collectively, these findings underscore the critical role of metabolic health in reducing the risk of EH and its progression toward endometrial cancer [52].

### 4.3. Genetic and Molecular Alterations

Somatic molecular alterations and inherited genetic conditions substantially contribute to EH pathophysiology and progression into cancer [53,54]. Lynch syndrome, also known as hereditary nonpolyposis colorectal cancer (HNPCC), associated with a markedly increased lifetime risk of endometrial cancer, frequently involves EH as a precursor lesion. A family history of endometrial cancer may similarly suggest inherited susceptibility [55].

Atypical EH exhibits molecular profiles that overlap with early endometrioid cancer, including alterations in PTEN and PI3K/AKT signaling, KRAS mutations, and early genomic instability. PTEN loss occurs in approximately 40–60% of atypical EH compared to 15–30% of non-atypical EH. KRAS mutations occur in roughly 10–20% of atypical EH, versus 5–10% in non-atypical lesions, while PI3K/AKT/mTOR pathway activation is observed more frequently in atypical EH/EIN, reflecting early oncogenic signaling [27,56]. Aberrant DNA methylation and occasional microsatellite instability are also common in atypical EH/EIN [27].

These molecular insights are presented in Table 1 and highlight that EH is influenced by intricate genetic and biochemical processes beyond hormonal imbalance. Identifying such changes has implications for risk stratification, surveillance of high-risk patients, and the development of biomarker-guided diagnostic and management strategies [57,58].

### 4.4. Inflammatory and Environmental Factors

Chronic low-grade inflammation, often associated with obesity, contributes to a pro-tumorigenic milieu through cytokine signaling and oxidative stress. Although there is currently little evidence, other environmental factors such as nutrition, a sedentary lifestyle, and substances that affect hormones have also been linked to dysregulation of estrogen pathways [62].

### 4.5. Interaction of Risk Pathways

These risk factors do not operate alone. For instance, insulin resistance and increased unopposed estrogen increase the risk for an obese woman with PCOS. These pathways may be further altered by genetic vulnerability, which would account for interindividual variation in the course of disease [63]. A comprehensive summary of the key risk factors for EH, their mechanisms, and clinical relevance is presented in Table 2.

### 4.6. Crosstalk Between Metabolic, Hormonal, and Genetic Pathways

Emerging evidence highlights extensive crosstalk between metabolic dysfunction, hormonal imbalance, and genetic susceptibility in the development of EH [68,69]. Obesity-induced insulin resistance results in chronic hyperinsulinemia that activates the insulin-like growth factor-1 (IGF-1) signaling pathway, stimulating PI3K/AKT and MAPK pathways independently of estrogen. Elevated IGF-1 and decreased IGF-binding proteins are observed in both obesity and PCOS, suggesting shared mechanisms linking metabolic and reproductive endocrine disorders to endometrial overgrowth [70].

Epigenetic alterations further integrate these processes. Obesity and PCOS are associated with global DNA hypomethylation, altered histone acetylation, and differential methylation of genes regulating steroidogenesis, inflammation, and cell-cycle control. These modifications enhance estrogenic signaling, reduce progesterone responsiveness, and potentiate oncogenic pathways such as PTEN/PI3K/AKT and KRAS, thereby increasing susceptibility to atypical EH/EIN in genetically predisposed women [71].

This interconnected network of metabolic, hormonal, and epigenetic influences may explain why women with similar clinical risk factors can experience markedly different disease courses. Understanding these interactions offers opportunities for future biomarker development and may support personalized preventive and therapeutic strategies.

## 5. Clinical Presentation and Diagnosis

EH has substantial clinical relevance since it can lead to endometrial cancer and often presents with abnormal uterine symptoms, it. To maximize patient results, it is essential to comprehend its appearance, diagnostic difficulties, and management techniques [72].

### 5.1. Clinical Presentation

AUB is the most common presenting symptom of AUB [73]. Menorrhagia, oligomenorrhea, or intermenstrual spotting are some of the symptoms that premenopausal women may experience, while postmenopausal women should be evaluated for even one vaginal bleeding episode [74,75]. Asymptomatic cases are occasionally discovered by chance when an endometrial sample or imaging is performed for other purposes. Suspicion should be heightened due to risk factors such as obesity, PCOS, or exposure to unopposed estrogen [27].

### 5.2. Imaging Modalities

Transvaginal ultrasonography (TVUS) is recommended first-line imaging modality, with reported sensitivity of 80–90% and specificity of 85–90% for detecting EH. Although the results are not pathognomonic, increased endometrial thickness, focal lesions, or abnormal endometrial echogenicity may raise suspicion [76]. Localized hyperplasia or polyps are examples of focused abnormalities that can be better detected with saline infusion sonohysterography (SIS). Although it is typically used for complex or ambiguous situations, magnetic resonance imaging (MRI) provides improved soft-tissue contrast and can help distinguish EH from cancer [77,78].

### 5.3. Histopathologic Evaluation

Histopathologic assessment is required for a definitive diagnosis. Office-based endometrial biopsy techniques (e.g., Pipelle) are minimally invasive but may yield insufficient tissue or miss focal pathology. Although it might overlook isolated lesions, D&C is still a diagnostic possibility [79,80]. The most accurate diagnosis is possible with a hysteroscopically guided biopsy since it enables a targeted sampling under direct visualization. The ability to differentiate between simple and complex hyperplasia, with or without atypia, is still based on histology and is used to guide prognosis and management [81]. A comparison of the diagnostic performance and appropriate use of different endometrial sampling techniques is summarized in Table 3.

### 5.4. Molecular and Biomarker Approaches

To enhance risk stratification, new molecular diagnostics are being investigated. EH may exhibit alterations in PTEN, KRAS, and PIK3CA, as well as aberrant DNA methylation and microsatellite instability, which may correlate with progression risk [84,85]. Immunohistochemical markers including p53, Ki-67, and mismatch repair (MMR) proteins are also under investigation. These methods have the potential to include molecular profiles into common diagnostic algorithms, even if they are not yet incorporated into routine clinical practice [86,87]. The diagnostic performance of TVUS, SIS, MRI, and biopsy-based methods is outlined in Table 4.

### 5.5. Differential Diagnosis

The differential diagnosis of EH includes endometrial polyps, endometritis, retained products of conception, and endometrial cancer. To guarantee accurate diagnosis and suitable treatment, a careful correlation between clinical history, imaging, and histology is necessary [94].

## 6. Classification and Histological Subtypes

It is crucial to accurately classify EH since the histological subtype directly influences therapy techniques and has a strong correlation with the risk of developing cancer. As EH is now recognized as a spectrum of proliferative abnormalities and a precursor to endometrial cancer, several classification systems have been developed over the past decades [1,95,96].

### 6.1. WHO 1994 Classification

One of the most extensively used frameworks is the WHO 1994 system. it classifies EH into four groups: simple hyperplasia without atypia, complex hyperplasia without atypia, simple atypical hyperplasia, and complex atypical hyperplasia [97]. Simple hyperplasia without atypia features mild glandular crowding and moderate irregularity, with a low potential for malignancy. In contrast, complex atypical hyperplasia is linked to a significantly higher risk of malignant transformation, reported in up to 30% of cases, and exhibits nuclear atypia along with architectural distortion. Despite its historical utility, the WHO 1994 classification has been criticized for poor interobserver reproducibility and significant challenges in reliably distinguishing simple from complex subtypes [64,98].

### 6.2. EIN System

The EIN system was developed to improve diagnostic precision than the older WHO 1994 system. EIN defines a clonal, premalignant lesion based on a combination of morphometric features, histologic criteria, and the exclusion of benign mimics. Diagnostic criteria include (i) a lesion size ≥ 1 mm, (ii) cytologic demarcation from the background endometrium, (iii) glandular crowding with reduced stromal volume, and (iv) exclusion of entities such as polyps or reparative changes [4].

EIN correlates strongly with concurrent or future endometrioid cancer, with progression risks of 25–40%. Compared with atypical hyperplasia in the WHO 1994 system, EIN demonstrates higher interobserver reproducibility and more accurate prediction of malignant potential [4].

For this reason, the WHO 2014 revision consolidated atypical hyperplasia and EIN into the unified category “atypical hyperplasia/EIN”, reflecting their shared biological significance while acknowledging that EIN is conceptually distinct from atypical hyperplasia as originally defined.

### 6.3. WHO 2014 Revision

Atypical hyperplasia/EIN and hyperplasia without atypia are the two main categories under which the WHO 2014 revision streamlined the classification, acknowledging these benefits. Much of the uncertainty included in the four-tiered 1994 method is removed by this two-tiered approach, which highlights the presence or absence of cytologic atypia as the most therapeutically significant aspect [97].

### 6.4. Clinical Implications

This classification change has immediate treatment ramifications from a clinical standpoint. Progestin therapy and close monitoring are frequently effective ways to manage hyperplasia without atypia, which has a comparatively low malignant potential—less than 5% of cases proceed to cancer [97]. Atypical hyperplasia/EIN, on the other hand, is now generally considered a premalignant lesion. For women who do not want to become pregnant in the future, a hysterectomy is the best course of action because it often coexists with or develops into endometrioid cancer [28]. Fertility-saving techniques may be taken into consideration for younger patients who want to maintain their fertility, but they need strict follow-up and careful observation for the persistence or advancement of the disease [99].

## 7. Management Strategies

### 7.1. Medical Therapy

Medical management it the cornerstone of treatment for EH without atypia and for atypical hyperplasia/EIN in women seeking for fertility preservation [100]. The primary substances employed are progestins, which inhibit unopposed estrogen activation and have antiproliferative effects. They may be administrated orally (e.g., megestrol acetate, medroxyprogesterone acetate), through depot formulations, or through an intrauterine system that releases levonorgestrel (LNG-IUS). The LNG-IUS has proven to be the most effective of these approaches, showing significant action in atypical lesions and regression rates of over 90% for hyperplasia without atypia [101]. Although adherence problems and systemic side effects might limit their use, oral progestins are still an effective alternative. Typically, endometrial sampling is performed at regular intervals to track the histologic response over the 6–12-month course of treatment [102].

### 7.2. Lifestyle Modification

Treatment results are improved by lifestyle and metabolic interventions, particularly for women who have metabolic syndrome or obesity. In addition to lowering the risk of recurrence, weight loss, improved insulin sensitivity, and optimized glycemic control may also enhance response to progestin therapy. Therefore, it is advised that a comprehensive treatment plan include nutritional advice, exercise, and medical management of comorbidities [103,104].

### 7.3. Surgical Management

When medicinal treatment fails or a woman with atypical hyperplasia/EIN has finished childbearing, surgery is still the only option. In these cases, the gold standard is a hysterectomy [105]. For postmenopausal women or those with elevated risk of ovarian cancer, bilateral salpingo-oophorectomy may be performed concurrently. Due to their reduced morbidity and quicker recovery, minimally invasive procedures such as robotic or laparoscopic hysterectomy are recommended. Crucially, it is not recommended to perform endometrial ablation since it complicates subsequent surveillance and may obscure persistent or recurrent condition [106].

Sentinel lymph node (SLN) mapping may be considered during hysterectomy in selected patients with atypical hyperplasia/EIN. Since 30–45% of women with this diagnosis are found to have concurrent cancer on final pathology, some centers offer SLN mapping to evaluate for occult nodal involvement, particularly when preoperative imaging raises suspicion or when individual cancer is elevated. Routine SLN assessment is not universally recommended for all cases, but it represents a reasonable individualized option in higher risk cases [107,108].

### 7.4. Fertility-Sparing Approaches

Conservative treatment with high-dose oral or intrauterine progestins may be an option for young women with atypical hyperplasia/EIN who want to maintain their fertility [109]. Endometrial biopsies must be performed on these patients every three to six months until histologic regression is confirmed. To minimize time away from therapy, conception should be tried as soon as remission is reached, frequently with the use of assisted reproductive technology. After childbearing is finished, a final hysterectomy is advised due to the possibility of recurrence [110,111].

### 7.5. Emerging and Investigational Therapies

Novel therapeutic options are under investigation for patients who do not respond adequately to conventional treatment. These include selective progesterone receptor modulators (SPRMs), aromatase inhibitors, and agents that target the PI3K/AKT/mTOR signaling pathway [112,113]. Early phase studies of SPRMs such as ulipristal acetate have reported histologic regression rates of 60–70% over approximately 12 weeks of therapy, with minimal adverse effects in small cohorts of 30–50 participants. Aromatase inhibitors, including letrozole, have been evaluated primarily in postmenopausal women or those with obesity-related EH, demonstrating reductions in endometrial thickness and symptomatic improvement in small studies of 20–40 patients; however, long-term safety data remain limited. Targeted therapies directed at the PI3K/AKT/mTOR pathway have shown antiproliferative effects in preclinical and early clinical studies, but further research is required to determine their safety profile, optimal dosing, and clinical efficacy [114]. Larger randomized trials will be essential to validate these emerging therapeutic options and define patient populations most likely to benefit.

### 7.6. Surveillance and Follow-Up

Regardless of the initial treatment strategy, ongoing surveillance is essential. Patients on medical treatment should undergo repeat endometrial sampling every three to six months until remission is confirmed [115]. Subsequently, annual follow-up evaluation is recommended. Although imaging modalities such as TVUS can offer additional information, histologic confirmation remains the definitive method for assessing disease status and must guide clinical decision-making [116,117].

## 8. Molecular Insights and Emerging Therapies

### 8.1. Molecular Pathogenesis

Recent research has shed insight on the molecular landscape of EH, emphasizing the genetic and epigenetic alterations that drive the development of cancer. PTEN inactivation is the most frequently observed change, contributing to PI3K/AKT pathway activation and increased cellular proliferation [118]. PIK3CA and KRAS mutations are also reported in a subset of lesions and further promote dysregulated signaling and clonal growth [119].

### 8.2. Biomarkers for Risk Stratification

Recent Novel molecular markers can be used in clinical settings to identify lesions that are more likely to progress. Genomic stability and proliferative activity can be predicted by immunohistochemical investigations of the proteins Ki-67, p53, and mismatch repair (MMR) [120]. Particularly in situations that are uncommon or borderline, the combination of histology and genetic profile may improve prognostic accuracy and direct customized treatment [121].

### 8.3. Targeted and Emerging Therapies

Gaining a better knowledge of molecular circuits has spurred research into targeted treatment approaches. Progestin receptor modulators (SPRMs), aromatase inhibitors, and medicines that disrupt the PI3K/AKT/mTOR pathway are being studied, especially for individuals who are not responding to conventional progestin medication. Personalized treatment may be possible with these treatments, which could increase effectiveness and lessen systemic side effects [122,123].

### 8.4. Translational Implications

Molecular results also impact preventative and early intervention strategies. The likelihood of developing cancer may be reduced by identifying patients with specific genetic alterations or high-risk molecular profiles, which would allow for targeted surveillance and timely intervention. As more accessible and standardized tests become available, it is expected that the current lack of integration of molecular testing into mainstream clinical practice will change [124,125,126]. A summary of the molecular alterations, biomarkers, and emerging therapeutic approaches in EH is provided in Table 5.

## 9. Prevention and Public Health Implications

Early detection, population-level initiatives, and modifiable risk factors must all be addressed in order to prevent endometrial EH and its development into endometrial cancer. Lifestyle treatments are crucial because obesity, metabolic syndrome, and unopposed estrogen exposure are among the major avoidable causes of EH [129,130]. For women who are at risk, controlling weight through diet, exercise, and the management of concomitant conditions like diabetes and hypertension can improve outcomes and lessen estrogen-driven endometrial growth.

Another essential component of prevention is hormonal control. The incidence of EH can be significantly reduced by avoiding unopposed estrogen therapy in postmenopausal women and encouraging progesterone-containing regimens when needed [131,132]. Additionally, ovulation-inducing treatments and cyclic progestin administration may help women with PCOS or chronic anovulation by counteracting prolonged estrogen exposure [133,134].

Rapid examination and action can be facilitated at the community level by greater knowledge of irregular uterine bleeding as an early warning indicator, especially in high-risk groups. Although universal endometrial screening is not yet advised, screening methods may be taken into consideration in some high-risk groups, such as women with Lynch syndrome. Targeted preventative interventions can also be supported by genetic counseling and testing for hereditary cancer disorders [135,136].

Reducing the burden of EH requires public health strategies that incorporate lifestyle modification programs, education, and access to healthcare services. The incidence of EH may be reduced, and the development of endometrial cancer may be avoided with early identification of at-risk patients and customized therapies [137,138].

## 10. Conclusions

EH represents a spectrum of proliferative abnormalities ranging from benign overgrowth to premalignant lesions with substantial potential for progression to endometrial cancer. Identification of high-risk groups and the direction of preventative and treatment methods are made possible by the identification of important risk factors, such as unopposed estrogen exposure, obesity, metabolic dysfunction, reproductive history, and genetic predispositions.

Accurate histopathologic classification remains central to clinical decision-making. From conservative progestin medication and fertility-sparing management to definitive hysterectomy in high-risk situations, the current two-tiered strategy that differentiates between atypical hyperplasia/EIN and hyperplasia without atypia guides decision-making. Patient outcomes are improved, and the risk of progression is decreased when early detection is achieved by combining clinical evaluation, imaging, endometrial sampling, and newly discovered molecular markers.

Although expanding molecular and genetic insights, such as alterations in PTEN, KRAS, and PI3K/AKT pathway activity, have depended on our understanding of EH biology, their clinical utility remains limited. These findings have not yet been translated into standardized diagnostic or therapeutic algorithms, and in some cases introduce additional complexity rather than actionable guidance. While molecular stratification may eventually enable more precise risk prediction and targeted therapy, current evidence remains preliminary. Robust validation through large, prospective studies will be essential before these approaches can be incorporated into routine clinical practice.

## Figures and Tables

**Table 1:** Frequency of key molecular alterations in EH subtypes.

Molecular Alteration	Mechanistic Role	Frequency in Non-Atypical EH	Frequency in Atypical EH/EIN
PTEN loss [59]	Tumor suppressor inactivation leading to PI3K/AKT pathway activation and uncontrolled proliferation	20–40%	55–85% [15]
KRAS mutation [19]	Activates RAS/MAPK signaling, promoting mitogenic drive	5–15%	10–30%
PI3K/AKT/mTOR pathway activation [60,61]	Enhances cell growth, survival, and proliferation via aberrant signaling	10–25%	35–50%

Abbreviations: EH: Endometrial Hyperplasia; EIN: Endometrial Intraepithelial Neoplasia.

**Table 2:** Summary of major risk factor categories contributing to EH, detailing underlying mechanisms and key clinical examples relevant to risk assessment and prevention.

Risk Factor	Mechanism	Examples	Clinical Relevance
Hormonal Influences [13,64]	Unopposed estrogen promotes proliferation Progesterone induces differentiation and inhibits mitosis	PCOS Estrogen-secreting tumors Tamoxifen Estrogen-only HRT Early menarche/late menopause Protective effects of OCPs and multiparity	Guides progestin therapy and ovulation induction
Metabolic Factors [65,66]	Obesity → increased aromatization Insulin resistance → enhanced mitogenic signaling Metabolic syndrome → pro-inflammatory state	obesity diabetes metabolic syndrome components	Highlights the importance of metabolic optimization for prevention
Genetic and Molecular Alterations [27]	Somatic and inherited mutations (e.g., PTEN, KRAS, PI3K/AKT/mTOR dysregulation) Promote uncontrolled proliferation	Lynch syndrome family history of endometrial cancer	Supports risk stratification, monitoring, and biomarker-guided therapy
Inflammatory and Environmental Factors [65]	Chronic inflammation and oxidative stress promote a tumorigenic milieu Lifestyle exposures can disrupt estrogen pathways	Obesity-related inflammation Sedentary lifestyle Diet Endocrine disruptors	Suggests preventive lifestyle modification
Interaction of Risk Pathways [67]	Synergistic effects between hormonal, metabolic, and genetic factors	Examples include obesity + PCOS + insulin resistance	Explains interindividual variation in disease progression

Abbreviations: EH: Endometrial Hyperplasia; PCOS; Polycystic Ovary Syndrome; OCPs: Oral Contraceptive Pills; HRT: Hormone Replacement Therapy.

**Table 3:** Comparison of commonly used endometrial biopsy techniques.

Technique	Advantages	Limitations	Best Used For
Pipelle biopsy [80]	Fast, office-based, inexpensive	May miss focal lesions; insufficient samples	Initial evaluation
D&C [82]	Broader sampling	Blind procedure; risk of missing focal disease	When office biopsy is inconclusive
Hysteroscopic biopsy [83]	Direct visualization; highest accuracy	Requires equipment and expertise	Suspicious focal lesions or atypia

**Table 4:** Diagnostic accuracy of imaging and tissue-based methods for evaluating suspected EH.

Diagnostic Method	Sensitivity (%)	Specificity (%)	Notes/Clinical Use
TVUS [88,89]	80–90	85–90	First-line test Assesses endometrial thickness and general morphology
SIS [90,91]	Higher than TVUS	Higher than TVUS	Best for evaluating focal lesions (polyps, localized hyperplasia)
MRI [92]	High for complex lesions	High	Reserved for indeterminate or suspicious cases
Pipelle biopsy [80]	70–90	98–100	Office-based May miss focal disease
Hysteroscopy (with biopsy) [93]	>95	>95	Gold standard for focal lesions and atypia detection

**Table 5:** Molecular Insights and Emerging Therapies in EH.

Category	Key Mechanisms	Clinical Relevance	Examples
Molecular Pathogenesis [127,128]	PTEN inactivation drives PI3K/AKT activation PIK3CA and KRAS mutations promote aberrant signaling Atypical lesions often show DNA methylation abnormalities and MSI	Provides mechanistic explanation for progression from EH to cancer Highlights interplay between hormonal and molecular drivers	PTEN loss KRAS mutation PIK3CA mutation MSI-high atypical lesions
Biomarkers for Risk Stratification [15]	IHC markers such as Ki-67, p53, and MMR proteins assess proliferative index and genomic stability	Identifies lesions with higher malignant potential and improves prognostic accuracy, especially in ambiguous cases	High Ki-67 index Abnormal p53 staining MMR deficiency indicating Lynch syndrome risk
Targeted and Emerging Therapies [54]	Development of SPRMs, aromatase inhibitors, and inhibitors of PI3K/AKT/mTOR pathways	Offers options for patients resistant to conventional therapy Enables personalized treatment with potentially reduced toxicity	Ulipristal acetate Letrozole mTOR inhibitors
Translational Implications [15,27]	Molecular profiling informs surveillance, early detection, and risk-adapted intervention strategies	Identifies high-risk patients and supports future integration of molecular diagnostics into routine care	Selection of patients for intensified surveillance Molecular-guided therapy selection.

## Data Availability

No new data were created or analyzed in this study. Data sharing is not applicable to this article.
